# Effects of Low and High Doses of Deoxynivalenol on Growth Performance, Blood Biochemistry, Histology, Metabolites, and Microbial Community in Adult Rats

**DOI:** 10.3390/biology14040429

**Published:** 2025-04-16

**Authors:** Jinyoung Jeong, Junsik Kim, Boram Lee, Cheolju Park, Minseok Kim

**Affiliations:** 1 Precision Animal Nutrition Division, National Institute of Animal Science, Wanju 55365, Republic of Korea; 2 Animal Biotechnology and Genomics Division, National Institute of Animal Science, Wanju 55365, Republic of Korea; 3 Division of Animal Science, College of Agriculture and Life Sciences, Chonnam National University, Gwangju 61186, Republic of Korea

**Keywords:** apoptosis, crop, deoxynivalenol, fibrosis, microbiome, mycotoxin, rat

## Abstract

Deoxynivalenol (DON) is a widespread mycotoxin which contaminates several crops, including maize, wheat, and barley. In this study, we investigated the effects of orally administered DON on growth performance, blood biochemistry, histology, the gut microbiome, and metabolism in rats. Six-week-old rats, acclimatized for one week, were subjected to different dietary treatments for 42 days, as follows: CON (control): 0.9% saline; T1: 0.5 ppm DON; T2: 50 ppm DON; and T3: 100 ppm DON. The T3 group had the lowest final body weight (298.5 ± 3.69 g) and average daily gain compared with the control group (338.9 ± 6.43 g, *p* < 0.05). The feed conversion ratio was highest in the T3 group (4.28 ± 0.28) compared with that in the control group (3.12 ± 0.13, *p* < 0.05). DON treatment significantly reduced serum levels of creatinine, amylase, urea nitrogen, and alkaline phosphatase, but not alanine aminotransferase. Fibrosis and apoptosis were exacerbated in various tissues with increasing DON concentration. The metabolite profiles of several tissues were significantly different in the DON-treated and control groups. In the cecum, DON treatment increased the abundance of Desulfobacteria, while decreasing that of Firmicutes. Our results indicate that DON levels above the maximum residue limit have serious health consequences for animals.

## Simple Summary

Deoxynivalenol (DON), a type B trichothecene produced by *Fusarium* species, is one of the most widespread mycotoxins, and contaminates various crops, such as maize, wheat, and barley. Food or feed contaminated with DON can cause a variety of health problems in humans and animals, including growth retardation, immune dysfunction, and dysbiosis, and can have a serious impact on public health. This study was conducted to evaluate the adverse effects of dietary intake of different doses of DON, from low to high, on rats. Our results indicate that DON toxicity in rats led to reduced growth performance and alterations in blood biochemistry in a dose-dependent manner. DON exposure also resulted in pronounced dose-dependent histological changes, including fibrosis and apoptosis, throughout the body. Microbial communities and metabolites in multiple organs were significantly affected by DON toxicity, with the effects becoming more pronounced at higher doses. This study demonstrates that DON toxicity is detrimental at doses ranging from below the maximum residue limit to high concentrations. The fundamental data obtained in this study might be useful in establishing criteria for determining DON contamination in feed and food.

## 1. Introduction

Deoxynivalenol (DON), a type B trichothecene produced by *Fusarium culmorum* or *Fusarium graminearum*, is one of the most widespread mycotoxins, contaminating a variety of crops, including maize, wheat, and barley [1,2]. Several studies have indicated that DON-contaminated foods contribute to various adverse human and animal health outcomes. Despite this, DON contamination in food and feed remains widespread, posing significant public health risks [3]. In the European Union, DON contamination has been found in 75.2% of feed samples and 43.5% of food samples [4], while in the United States, it has been detected in 64.1% of feed samples and 65% of multigrain foods [5,6]. DON contamination has been reported in 96.4% of feed samples in Asia, in 98% of cereal-based products in China, and in 95% of 494 feed samples in South Korea [6,7,8].

Previous studies have shown that DON causes multiple toxic effects throughout the animal body [9]. Ingestion of DON can lead to a variety of adverse effects, ranging from vomiting, diarrhea, gastroenteritis, and immune dysfunction to more severe consequences, such as leukopenia, endotoxemia, shock, and death [10]. Growth retardation is a representative effect of DON toxicity, and contaminated food can cause acute symptoms, such as vomiting, diarrhea, fever, and abdominal pain, which may lead to loss of appetite and reduced productivity in livestock farming [11,12]. In addition, DON toxicity induces reactive oxygen species (ROS) production, which leads to lipid peroxidation and an imbalance in the intracellular antioxidant system [12]. This can ultimately lead to histopathological abnormalities, such as fibrosis and apoptosis, as well as dysbiosis of the gut microbiota [13]. DON toxicity also negatively affects various metabolic processes, including glycolysis, gluconeogenesis, sucrose metabolism, galactose metabolism, oxidative metabolism, and glutaminolysis [14]. These toxic effects can vary widely depending on the dose of DON. For example, low chronic doses cause anorexia, immune dysfunction, and reproductive problems. High acute doses cause vomiting, leukocytosis, circulatory shock, and death [15].

DON toxicity depends on differences between species in its metabolism, absorption, distribution, and excretion [10]. In general, the susceptibility of different species to DON present in feed varies in the following order: pigs > mice > rats > poultry ≈ ruminants [16]. The rat model provides a practical approach to study the effect of toxicity on biological systems, as it allows researchers to conduct analyses over the entire lifespan of the animal [13]. In addition, understanding the toxicokinetics and metabolism of DON using this model can provide valuable information for risk assessment, prevention, and control [2]. Several studies evaluating the effects of DON toxicity in rats have shown that growth performance varies considerably with the dose of DON. In a previous study, we observed that oral administration of DON at 0.02 and 0.2 mg/L to rats resulted in a 5.9% reduction in final body weight at the 0.2 mg/L dose, with no difference in weight gain or feed intake [13]. Sprando et al. [17] exposed male rats to various concentrations of DON (0, 0.5, 1, 2.5, and 5 mg per kg) via gastric intubation for 28 days, and found that the 2.5 mg/kg treatment group had a 23.1% reduction in body weight gain, whereas the 5 mg/kg group had a 13.6% reduction in final body weight and a 19.1% reduction in feed intake. Similarly, Collins et al. [18] orally administered DON (0, 0.5, 1, 2.5, or 5 mg/kg bw) once daily for 20 days to pregnant female rats. The 5 mg/kg treatment group showed a 21.1% reduction in food consumption and a 59.5% reduction in body weight gain compared with the control.

This study examined the effect of different doses of DON, from low to high, on growth performance, hematological, intestinal microbiological, histopathological, and metabolite parameters in rats by orally administering 0.5, 50, or 100 ppm DON. We also performed correlation analyses between body weight and these parameters to identify potential biomarkers of DON toxicity.

## 2. Material and Methods

### 2.1. Ethics Statements

All experimental procedures were reviewed and approved by the National Institute of Animal Science, Korea Institutional Animal Care and Use Committee (No. NIAS-2022-0546).

### 2.2. Animals and Study Design

The male rats (Sprague Dawley) used in this study were obtained from Koatech (Pyeongtaek, Republic of Korea). A total of 32 SD rats were kept in individual cages (27.7 cm wide × 42.3 cm long). After one week of acclimation, the light/dark cycle (12 h:12 h), temperature (22 ± 1 °C), and relative humidity (55 ± 5%) were maintained for the entire experimental period using an air conditioning system. Animals were randomly divided into four groups of eight animals each: (1) the control (CON) group, fed a basal diet; (2) the T1 group, fed a basal diet + 0.5 mg/L DON; (3) the T2 group, fed a basal diet + 50 mg/L DON; and (4) the T3 group, fed a basal diet + 100 mg/L DON. We established experimental groups to assess in vivo concentration changes and their correlation with observed effects in rats, as described previously [13]. The experiment was blinded to the greatest extent possible to ensure objectivity of the results. DON (TripleBond, Guelph, ON, Canada) was combined with an organic solvent (95% ethyl alcohol; Lab Alley, Austin, TX, USA) as needed. For 42 days, animals were administered either 0.9% saline or DON dissolved in 0.9% saline daily. The rats were given food and water ad libitum, and were anesthetized using CO_2_. Blood and tissues, including the liver, kidney, muscle, jejunum, and abdominal fat, were collected rapidly. Tissues, cecal contents, and feces were immediately frozen in liquid nitrogen and stored at −80 °C (UniFreez U500, Daehan Scientific, Wonju, Republic of Korea). Growth characteristics, including the average daily gain (ADG), average daily feed intake (ADFI), feed conversion ratio (FCR), and average body weight change, were calculated based on a previous study [13]. The Yamane formula was used to calculate the sample size.

### 2.3. Blood Biochemical Analysis

Blood samples were taken from each rat via cardiac puncture on the 42 days of the experiment and placed in tubes containing no anticoagulant. Serum was separated by centrifuging the blood at 700× *g* for 15 min at 4 °C (VS-550, Vision Scientific Co., Daejeon, Republic of Korea), and then stored at −80 °C. A VetTest chemistry analyzer (IDEXX, Westbrook, ME, USA) was employed to evaluate a range of blood parameters: glucose, creatinine (CREA), blood urea nitrogen (BUN), phosphate, calcium, total protein, albumin, globulin, alanine aminotransferase (ALT), alkaline phosphatase (ALKP), total bilirubin, cholesterol, amylase (AMYL), and lipase.

### 2.4. Histological Analysis

For histological analysis, tissues were fixed in 10% neutral buffered formalin (NBF; Sigma-Aldrich, St. Louis, MO, USA). Tissue samples (0.5 cm × 0.5 cm) from the kidney, liver, jejunum, muscle, and adipose tissue were collected from each rat on day 42 of the experiment. The fixed specimen was dehydrated by passing through an ascending series of ethanol (70–100%, Sigma-Aldrich, Steinheim, Germany), cleared in xylene (Sigma-Aldrich), and embedded in paraffin. The embedded samples were sectioned at 5 μm thickness and heated on a slide warmer at 45 °C for 3 h (77 Slide Warmer, Fisher Scientific, Waltham, MA, USA). The sections were deparaffinized in xylene, rehydrated by passing through a descending series of ethanol (100–70%), and rinsed in distilled water. The sections were then stained using Masson’s trichrome staining and an in situ cell death detection kit for fibrosis and apoptosis, respectively. The stained slides were examined under a microscope (Micrometrics; Nikon ECLIPSE E200, Tokyo, Japan) at 200× magnification. The area (%) of collagen fiber positive for Masson’s trichrome and apoptotic cell staining was quantified using the ImageJ software (ver. 1.54).

### 2.5. DNA Preparation and Microbial Sequencing of Cecal and Fecal Contents

Bacterial DNA was extracted using the bead-beating-plus-column method [19] and QIAamp DNA kit (Qiagen, Germany). Qualitative and quantitative analysis of the extracted DNA was performed via electrophoresis on a 1% agarose gel and using a microplate reader (Infinite M NANO, Tecan, Republic of Korea), respectively. Samples were prepared for sequencing on the PacBio instrument as per the Single Molecule Real-Time (SMRT) Bell Template Preparation Guide. SMRT bell libraries were constructed by ligating adapters to the DNA ends, and then annealing sequencing primers and polymerase to the library for SMRT sequencing, using the Sequel II Binding Kit 2.1 and Sequel II DNA Internal Control Complex 1.0 (PacBio, Menlo Park, CA, USA). For 16S rRNA bacterial sequencing, primers 27F (5′-AGRGTTYGATYMTGGCTCAG-3′) and 1492R (5′-RGYTACCTTGTTACGACTT-3′) were used to amplify the full-length variable regions of the gene, creating an amplicon 1400 base pairs in length. PCR was performed for 25 cycles: 30 s at 95 °C, 30 s at 57 °C, and 30 s at 72 °C. Adapter controls, including the negative control, were then applied in eight cycles. The resulting amplicons were sequenced on an Illumina MiSeq platform (Illumina, San Diego, CA, USA) at Macrogen (Seoul, Republic of Korea) and analyzed using Quantitative Insights into Microbial Ecology (QIIME 2, Ver. 2021.8). An Agilent Technologies 2100 Bioanalyzer with a DNA 1000 chip was used to assess the size of PCR-enriched fragments. The cluster density in the prepared libraries was optimized using qPCR, according to Illumina guidelines, to ensure accurate data. The DADA2 statistics package (v1.20.0) was used to process the Illumina sequences. Primer sequences were first removed and reads were trimmed based on length. The reads with more than five expected errors were removed. The remaining reads were de-replicated and analyzed for sequencing errors using loessErrfun. True sequence variants were inferred. Forward and reverse reads were merged to produce complete denoised sequences. The SILVA v.138.1 database was used to identify and remove chimeric amplicon sequence variants (ASVs) and to annotate the remaining reads. Pacific Bioscience data were demultiplexed and circular consensus sequences were generated using the SMRT-Link analysis software (9.0). Nineteen high-fidelity passes were used. Quality control was then carried out using the R statistical software (ver. 4.4.2). Chimeric ASVs were removed and the remaining reads were annotated using a naive Bayesian classifier against a database of species. A 2% discrepancy is required for an optimal match. Taxonomic annotations were then used to generate contingency tables. To assess metabolic potential, 16S rRNA sequence reads were clustered into ASVs using QIIME2. The ASV table was imported into PICRUSt2, and the Kyoto Encyclopedia of Genes and Genomes (KEGG) database was used to predict the functional gene content of the Greengenes database of 16S rRNA gene sequences.

### 2.6. Preparation and Analysis of Metabolites in Different Tissues

For metabolite analysis, serum samples were treated with acetone, stirred, and refrigerated, before conducting supernatant extraction as described previously [13]. Briefly, after drying and reconstitution with methanol containing terfenadine as an internal standard, the solution was analyzed using UPLC-Q-TOF-MS (Waters, Milford, MA, USA). Liver, kidney, cecum, and feces samples were homogenized in methanol with terfenadine, and all prepared samples were injected into an Acquity UPLC BEH C18 column. Blood samples were processed within 12 min at 40 °C, while other samples required 16 min. Detection occurred via Q-TOF-MS in ESI mode, with data collected across a specific m/z range. Key parameters included optimized voltage settings and desolvation conditions. Leu-enkephalin ([M + H] = 556.2771) was analyzed every 10 s. Quality control samples, prepared by pooling all study samples, were run every 10 injections. MS/MS data were acquired in the collision energy range of 10–45 eV for m/z 50–1500. MarkerLynx 4.1 was used for data processing and calculating mass-to-charge ratios and ion intensities. It was also used for data acquisition and alignment, using peak–peak filtering, noise suppression, a 5% bandwidth, and a 10,000 minimum intensity threshold. Alignment parameters included a mass window of 0.05 Da and a retention time window of 0.2 min, with normalization to standards. ChemSpider, HMDB, METLIN, and the relevant literature were used to identify metabolites.

### 2.7. Statistical Analysis

SIMCA-P+ version 12.0.1 (Umetrics, Umeå, Sweden) was used for statistical analysis of LC-MS data. Partial least squares discriminant analysis (PLS-DA) was used to visualize the results, evaluated using the R2X, R2Y, Q2, and permutation tests. A permutation test was performed to cross-validate the PLS-DA results. Furthermore, relative metabolite abundances were analyzed using one-way analysis of variance (ANOVA) followed by Duncan’s test (*p* < 0.05) with SPSS 17.0 (SPSS Inc., Chicago, IL, USA). The linear discriminant analysis (LDA) effect size (LEfSe) was used to analyze the differential abundance of taxa among the four treatment groups (LDA score > 3). ANOVA with Tukey’s test, performed using the Prism statistical software (ver. 9.5.1, GraphPad Software, San Diego, CA, USA), was used to compare growth performance, biochemical analyses, and metabolites among the four treatment groups. The relationship between the final body weight and biochemical parameters and metabolites was analyzed using linear regression. Permutational multivariate analysis of variance (PERMANOVA) with PAST3 and 9999 random permutations was used to compare beta diversity and functional genetic profiles among the four treatment groups. The results are expressed as the mean ± the standard error of the mean (SEM). The statistical significance was set at *p* < 0.05, which indicated a significant difference between the control and treatment group.

## 3. Results

### 3.1. Effects of DON on Growth Performance and Tissue Weight

**Figure 1:**
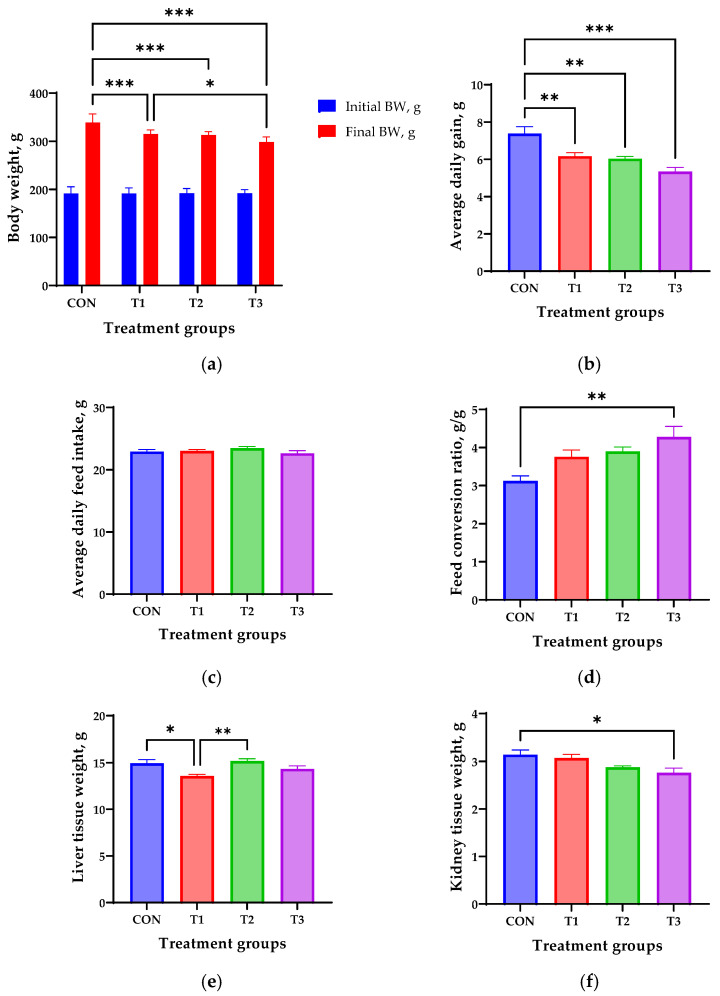
Growth performance and tissue weight in 7-week-old rats orally administered different doses of deoxynivalenol (DON) for 42 days. (**a**) Initial and final body weight (BW). (**b**) Average daily gain. (**c**) Average daily feed intake. (**d**) Feed conversion ratio. (**e**) Liver tissue weight. (**f**) Kidney tissue weight. Treatment groups: CON: control group, 0.9% saline by gavage; T1: 0.5 mg/L DON with 0.9% saline by gavage; T2: 50 mg/L DON with 0.9% saline by gavage; T3: 100 mg/L DON with 0.9% saline by gavage. * *p* < 0.05, ** *p* < 0.01, *** *p* < 0.001.

Figure 1 shows the effects of oral DON administration on growth performance and tissue weight in 7-week-old rats over a 42-day period. The initial body weight (191.7 ± 1.84 g) was not significantly different among the treatment groups, but the final body weight was significantly lower in all DON-treated groups than in the control group (*p* < 0.01, Figure 1a), and was lowest in the T3 group (298.5 ± 3.69 g). Similarly, the ADG was significantly decreased in all the DON treatment groups compared with that in the control group (*p* < 0.05, Figure 1b), and was lowest in the T3 group (5.35 ± 0.22 g). The ADFI was not significantly different among the treatment groups (Figure 1c). The FCR was significantly higher in the T3 group (4.28 ± 0.28) than in the control group (3.12 ± 0.13, Figure 1d). The liver and kidney weights were measured after oral DON administration. The liver weight of rats in the T1 group exhibited an 8.7% decrease compared with the weight in the control group (*p* < 0.05, Figure 1e). The kidney weight in the T3 group rats exhibited a 12.1% decrease compared with that in the control group (*p* < 0.05, Figure 1f).

### 3.2. Effects of DON on Blood Biochemistry

**Figure 2:**
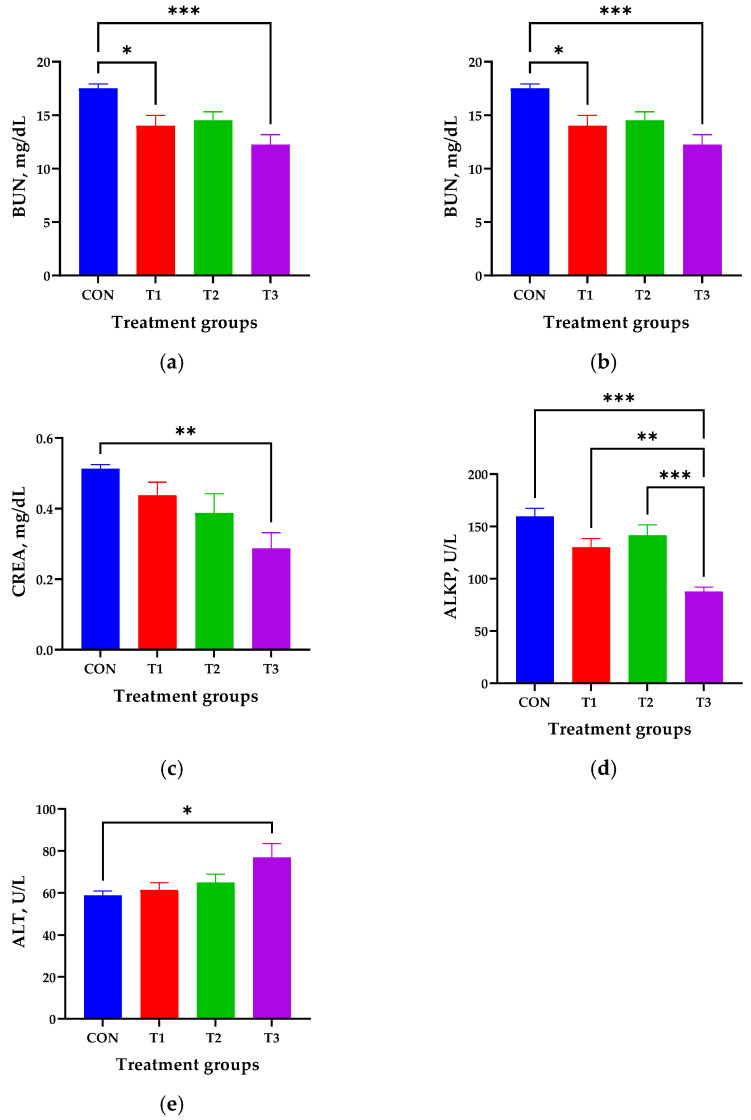
Blood biochemistry, including (**a**) amylase (AMYL), (**b**) blood urea nitrogen (BUN), (**c**) creatinine (CREA), (**d**) alkaline phosphatase (ALKP), and (**e**) alanine aminotransferase (ALT), in 7-week-old rats orally administered deoxynivalenol (DON) for 42 days. After 42 days of oral DON gavage, blood samples were collected from each rat via cardiac puncture. Treatment groups: CON: control group, 0.9% saline by gavage; T1: 0.5 mg/L DON with 0.9% saline by gavage; T2: 50 mg/L DON with 0.9% saline by gavage; T3: 100 mg/L DON with 0.9% saline by gavage. * *p* < 0.05, ** *p* < 0.01, *** *p* < 0.001.

The effects of oral DON administration for 42 days on blood biochemistry in 7-week-old rats are illustrated in Figure 2. Biochemical parameters that were not significantly different among dietary groups are not shown. The activities of AMYL (1847 ± 53.5) and ALKP (87.8 ± 4.31), and the concentrations of BUN (12.3 ± 0.92) and CREA (0.28 ± 0.04), were significantly lower in the T3 group than in the control group (*p* < 0.05, Figure 2a–d), whereas the ALT activity (76.8 ± 6.69) was significantly increased in the T3 group compared with that in the control group (*p* < 0.05, Figure 2e).

### 3.3. Effects of DON on Histological Changes

**Figure 3:**
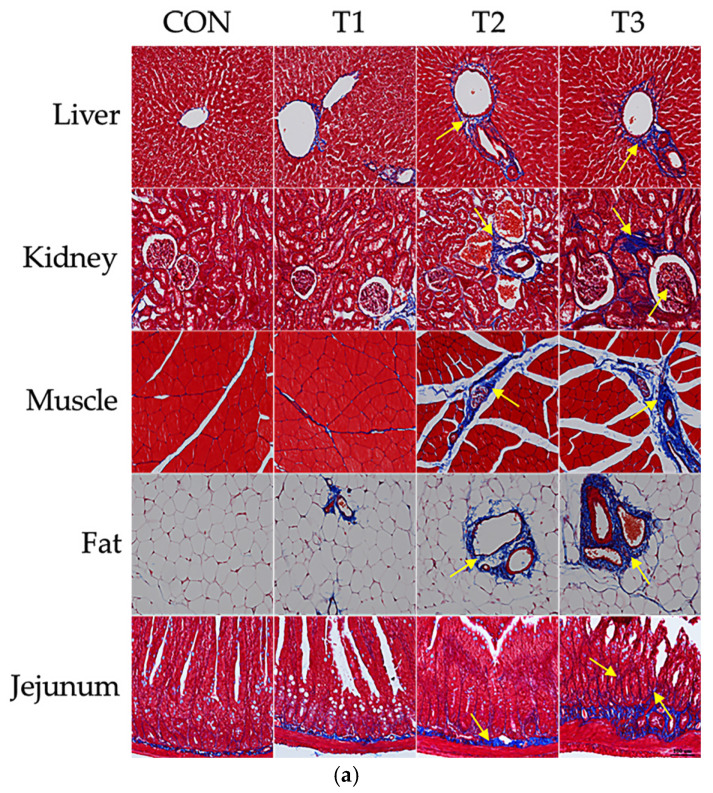

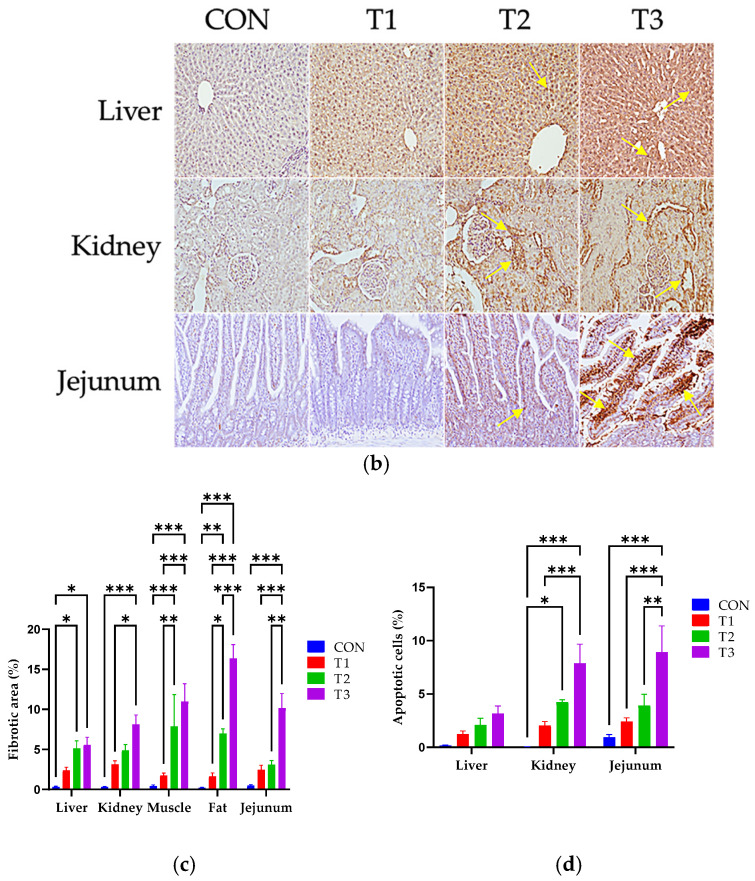
Histological changes in different tissues of 7-week-old rats orally administered different doses of deoxynivalenol (DON) for 42 days. (**a**) Histological images of the liver, kidney, muscle, jejunum, and adipose tissue sections subjected to Masson’s trichrome staining to detect fibrosis. (**b**) Apoptosis assessed using terminal deoxynucleotidyl transferase dUTP nick-end labeling (TUNEL) staining. Quantification of collagen (**c**) and apoptotic cell (**d**) staining was performed using the ImageJ software. The data are presented as the mean ± SEM. Statistical significance was considered for DON-treated groups compared with the control group by one-way ANOVA followed by Tukey’s post hoc test. Treatment groups: CON: control group, 0.9% saline by gavage; T1: 0.5 mg/L DON with 0.9% saline by gavage; T2: 50 mg/L DON with 0.9% saline by gavage; T3: 100 mg/L DON with 0.9% saline by gavage. The yellow arrows indicate positive signals. * *p* < 0.05, ** *p* < 0.01, *** *p* < 0.001.

Masson’s trichrome staining was performed to examine histopathological changes in the liver, kidney, muscle, jejunum, and adipose tissue (Figure 3a). The control group retained a relatively normal hepatic lobular structure. In contrast, in the T1 group, slight collagen deposition was observed around the portal vein compared with that in the control group, indicating the onset of mild fibrosis. In the T2 group, collagen deposition around the portal vein increased significantly and fibrous septa were formed, which became more severe in the T3 group. In the kidney, no evidence of fibrosis was noted in the control group, whereas in the T1 group, collagen deposition around the glomeruli was observed, which became more pronounced in the T2 group and extended into the tubular regions. In the T3 group, the degree of fibrosis was further increased. In the muscle tissue, the control group exhibited uniformly arranged and closely spaced muscle fibers. The muscle fibers in the T1 group appeared similar to those in the control group, although some areas showed increased collagen deposition. In both the T1 and T2 groups, there was minimal collagen deposition between the muscle fibers, indicative of an early stage of fibrosis compared with that in the control group. In contrast, in the T2 and T3 groups, spacing between the muscle fibers increased and collagen deposition around and between the fibers increased significantly, indicating advanced fibrosis. In the adipose tissue, the control group showed uniformly sized adipocytes with no signs of fibrosis, whereas the T1 group exhibited small amounts of collagen deposition around certain blood vessels. In the T2 group, the fibrous tissue surrounding the blood vessels became thicker and the overall collagen deposition within the adipose tissue increased. In the T3 group, collagen deposition around several blood vessels or structures increased significantly, indicating a more pronounced change in the adipose tissue structure. The control, T1, and T2 groups showed only a slight increase in collagen deposition in the submucosal area of the jejunum, with no major changes in the overall tissue structure. However, in the T3 group, blue-stained collagen deposition in the submucosal area was more pronounced, the overall barrier was thicker, and the villus shape was significantly deformed compared to the normal shape.

To evaluate apoptosis, TUNEL staining was performed on the liver, kidney, and jejunum (Figure 3b). Prominent apoptotic activity was detected in the apical regions of jejunal villi, along with renal cells and hepatocytes. Furthermore, the number of TUNEL-positive cells increased with increasing DON concentrations, indicating a dose-dependent increase in apoptosis compared with that in the control group.

Quantitative analysis of fibrosis from five different tissues (Figure 3c) and the apoptosis index in three different tissues (Figure 3d) are presented.

### 3.4. The Influence of DON on the Alpha and Beta Diversity of the Microbiome in Cecal and Fecal Samples

**Figure 4:**
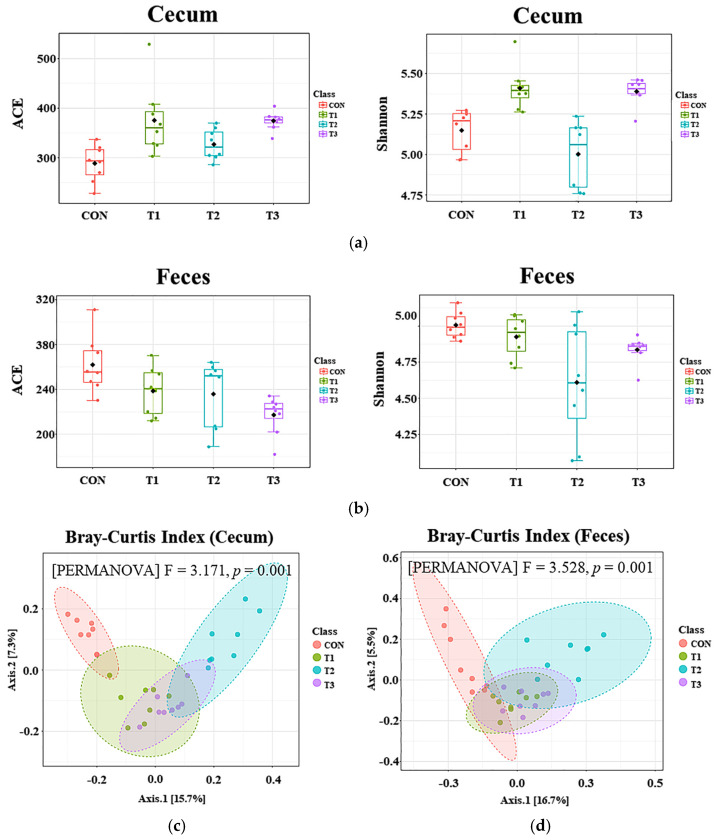
Analysis of alpha and beta diversity in 7−week-old rats orally administered different doses of deoxynivalenol (DON) for 42 days. Alpha diversity in the (**a**) cecum and (**b**) feces was assessed using the abundance-based coverage estimator (ACE) and Shannon indices. In the cecum, both the ACE and Shannon indices were increased in the DON treatment groups (*p* < 0.001). Conversely, in the feces, both indices were decreased in the DON treatment groups (*p* < 0.001). Beta diversity in the (**c**) cecum and (**d**) feces was analyzed using the Bray–Curtis index, revealing distinct shifts in microbiome composition. Significant differences (*p* = 0.001) were found among the dietary treatment groups in both cecal and fecal samples. Treatment groups: CON: control group, 0.9% saline by gavage; T1: 0.5 mg/L DON with 0.9% saline by gavage; T2: 50 mg/L DON with 0.9% saline by gavage; T3: 100 mg/L DON with 0.9% saline by gavage.

The effect of oral DON administration on the diversity of the cecal and fecal microbiota was assessed by measuring alpha diversity using the abundance-based coverage estimator (ACE) and Shannon indices (Figure 4). The ACE index is a measure of species richness or the number of species, with higher values indicating more species [20]. The Shannon index is used to measure species diversity, and is influenced by the species richness and community evenness of the sampled community [20]. In the cecum, the ACE and Shannon indices were higher in the DON treatment groups (*p* < 0.001, Figure 4a). Conversely, in the feces, the ACE and Shannon indices were lower in the DON treatment groups (*p* < 0.001, Figure 4b). Beta diversity, assessed using the Bray–Curtis index, revealed significant shifts in microbiome composition, with significant differences (*p* = 0.001) among the treatment groups for both cecal and fecal samples (Figure 4c,d).

### 3.5. Effects of DON on the Phyla of the Cecal and Fecal Microbiota

**Figure 5:**
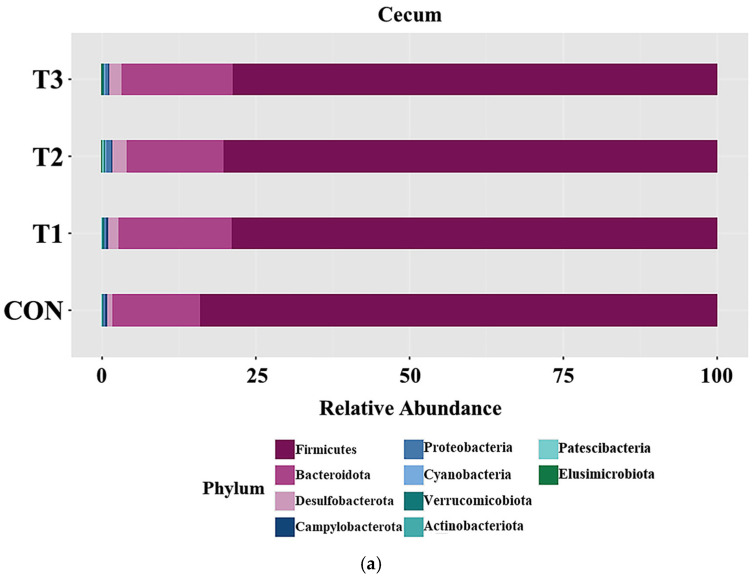

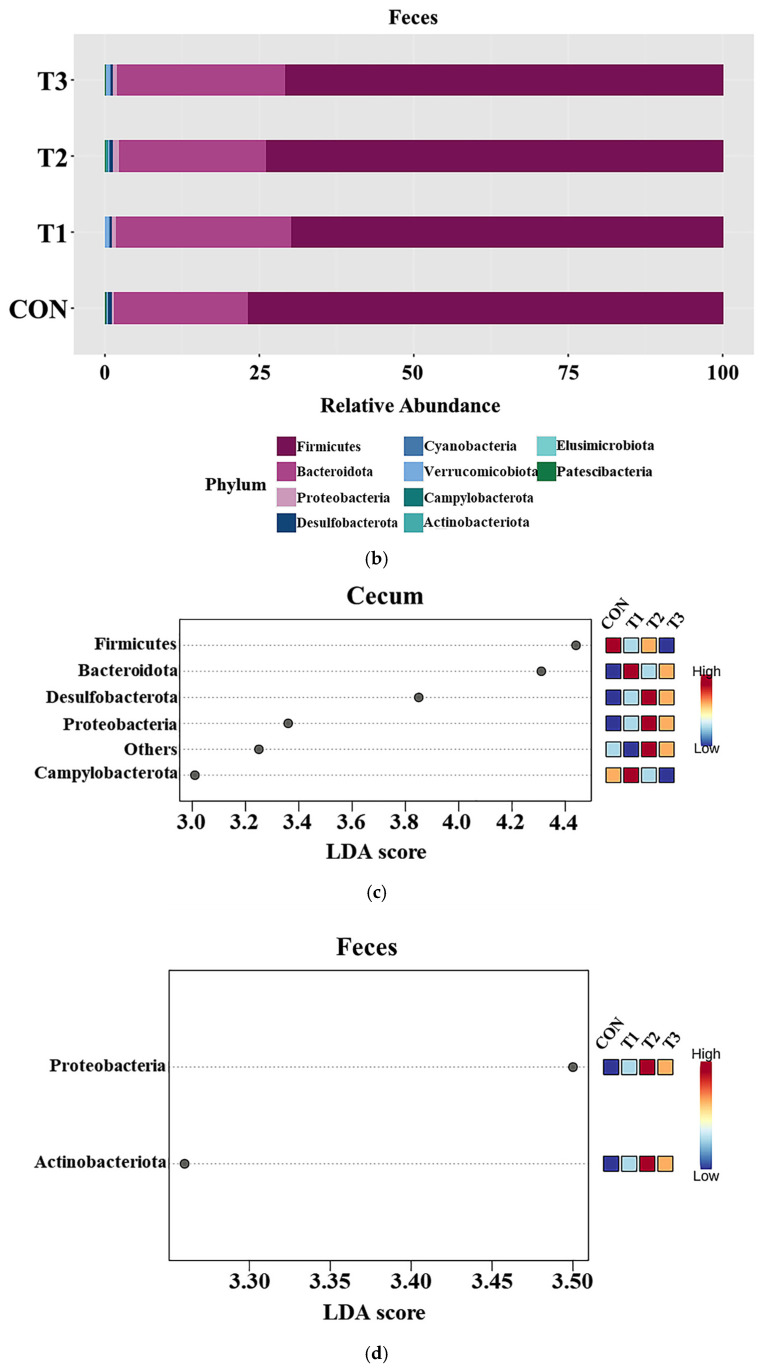
Microbial taxonomic bar plots and graphical representation of the linear discriminant analysis (LDA) effect size (LEfSe) at the phylum level for rats orally administered different doses of deoxynivalenol (DON) for 42 days. In (**a**) cecum and (**b**) feces, the taxonomic composition of the microbiota among all the dietary treatment groups was compared on the basis of relative abundance (taxon reads/total reads in cecum and feces). The horizontal bar on the graphs for (**c**) the cecum and (**d**) the feces is the log_10_ transformed LDA score. The relative abundance of the bacterial taxa was statistically significant (*p* < 0.05). Treatment groups: CON: control group, 0.9% saline by gavage; T1: 0.5 mg/L DON with 0.9% saline by gavage; T2: 50 mg/L DON with 0.9% saline by gavage; T3: 100 mg/L DON with 0.9% saline by gavage.

Figure 5 presents taxonomic bar plots illustrating the mean relative abundance of phylum levels in the cecum (Figure 5a) and feces (Figure 5b) across the treatment groups. Across the treatment groups, Firmicutes was the predominant phylum in both the cecum (81.27%) and feces (73.6%), with Bacteroidota following as the second most abundant (cecum: 16.14%; feces: 24.7%). To determine the distinct taxa among dietary treatment groups, LEfSe analysis was conducted at the phylum level (Figure 5c,d). In the cecum, the LEfSe analysis showed that Firmicutes (control group: 84.78%; T1: 79.66%; T2: 81.02%; T3: 79.27%) was relatively abundant in the control group, whereas Bacteroidota (control group: 13.73%; T1: 17.86%; T2: 15.33%; T3: 17.62%) was relatively rare. In the T2 and T3 groups, Desulfobacterota levels were higher (T2: 2.13%, T3: 1.87%) than in the control (0.73%) and T1 (1.35%) groups. Similarly, Proteobacteria levels were higher in T2 (0.55%) and T3 (0.40%) than in the other groups. In contrast, Campylobacterota levels were higher in the control (0.38%) and T1 (0.45%) groups than in T2 (0.26%) and T3 (0.24%). Fecal sample analysis revealed that the T2 group had the highest Proteobacterota levels, at 0.84%, while the other groups had lower levels. Additionally, Actinobacteriota were almost absent in the control group (0.00%), but increased to 0.02% in T1, 0.37% in T2, and 0.07% in T3.

### 3.6. Effects of DON on the Cecal and Fecal Microbiota of the Species

**Figure 6:**
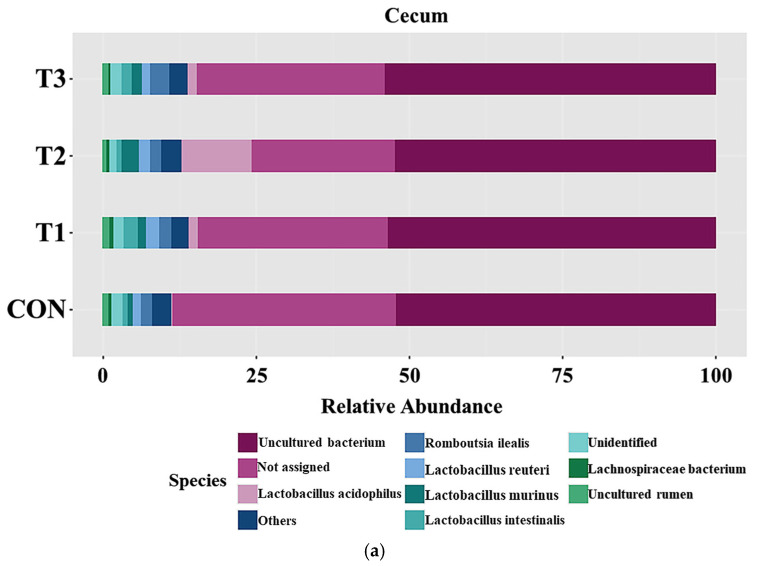

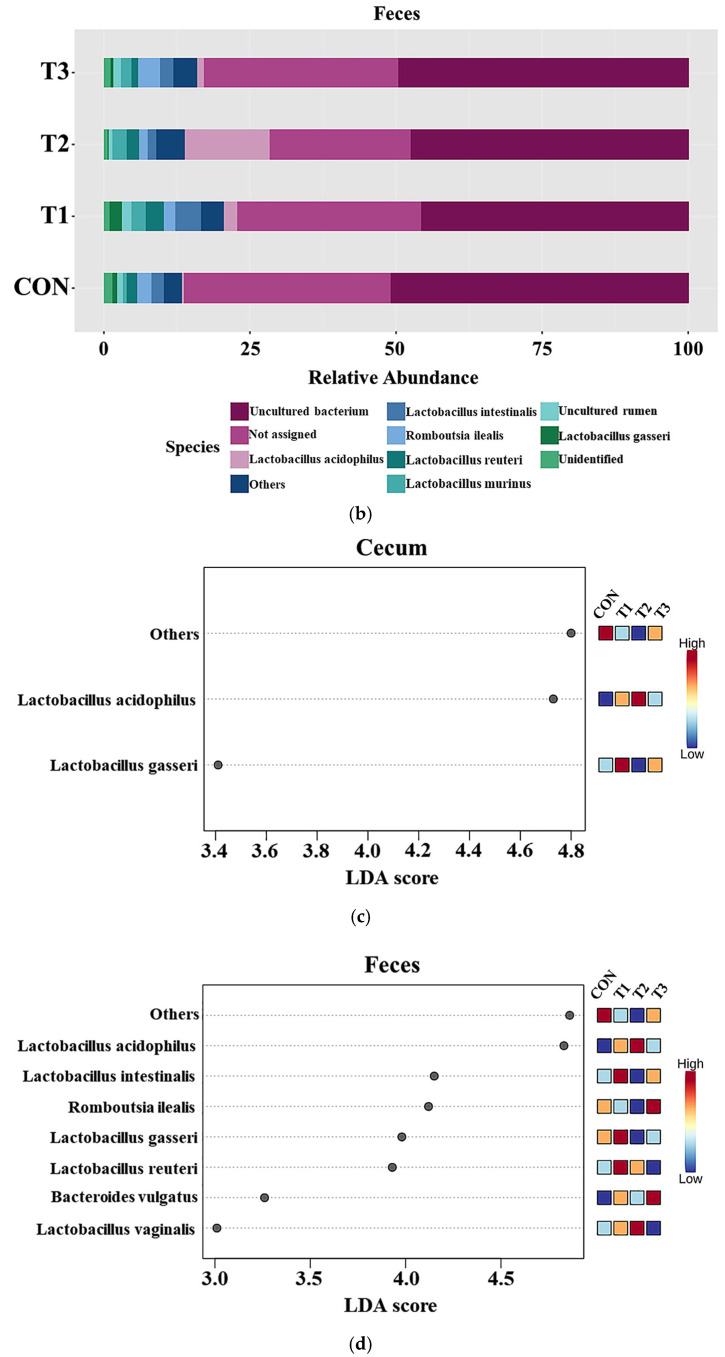
Microbial taxonomic bar plots and graphical representation of the linear discriminant analysis (LDA) effect size (LEfSe) at the species level for rats orally administered different doses of deoxynivalenol (DON) for 42 days. In (**a**) cecum and (**b**) feces, the taxonomic composition of the microbiota among all the dietary treatment groups was compared on the basis of relative abundance (taxon reads/total reads in cecum and feces). The horizontal bar on the graphs for (**c**) the cecum and (**d**) the feces is the log_10_ transformed LDA score. Bacterial taxa were statistically significant (*p* < 0.05) in terms of relative abundance. Treatment groups: CON: control group, 0.9% saline by gavage; T1: 0.5 mg/L DON with 0.9% saline by gavage; T2: 50 mg/L DON with 0.9% saline by gavage; T3: 100 mg/L DON with 0.9% saline by gavage.

Figure 6 presents taxonomic bar plots illustrating the mean species-level relative abundance in the cecum (Figure 6a) and feces (Figure 6b) across the treatment groups. In the cecum, *Lactobacillus acidophilus* (3.49%) was the most abundant species, followed by *Romboutsia ilealis* (2.05%), *Lactobacillus reuteri* (1.70%), *Lactobacillus murinus* (1.58%), and *Lactobacillus intestinalis* (1.58%). In the feces, *Lactobacillus acidophilus* (4.45%) was the most abundant species, followed by *Lactobacillus intestinalis* (2.41%), *Romboutsia ilealis* (2.39%), *Lactobacillus reuteri* (2.09%), and *Lactobacillus murinus* (2.09%). LEfSe was used to identify differentially abundant taxa at the species level (Figure 6c,d). In the cecum, the LEfSe analysis showed that *Lactobacillus acidophilus* (control group: 0.02%; T1: 1.33%; T2: 10.76%; T3: 1.19%) was least abundant in the control group. *Lactobacillus gasseri* (control group: 0.57%; T1: 0.67%; T2: 0.15%; T3: 0.57%) was least abundant in the T2 group. In the feces, *Bacreroides vulgatus* (control group: 0.34%; T1: 0.70%; T2: 0.62%; T3: 0.71%) and *Lactobacillus acidophilus* (control group: 0.09%; T1: 2.26%; T2: 13.74%; T3: 1.13%) were least abundant in the control group. *Lactobacillus gasseri* (control group: 0.81%; T1: 2.11%; T2: 0.21%; T3: 0.58%), *Lactobacillus intestinalis* (control group: 1.81%; T1: 4.18%; T2: 1.34%; T3: 2.05%), and *Lactobacillus reuteri* (control group: 1.58%; T1: 3.17%; T2: 2.00%; T3: 1.47%) were among the most abundant species in the T1 group. *Romboutsia ilealis* (control group: 2.33%; T1: 1.83%; T2: 1.18%; T3: 3.82%) had the highest abundance in the T3 group.

### 3.7. Effects of DON on the Metabolome Profiles of Blood and Tissue

**Figure 7:**
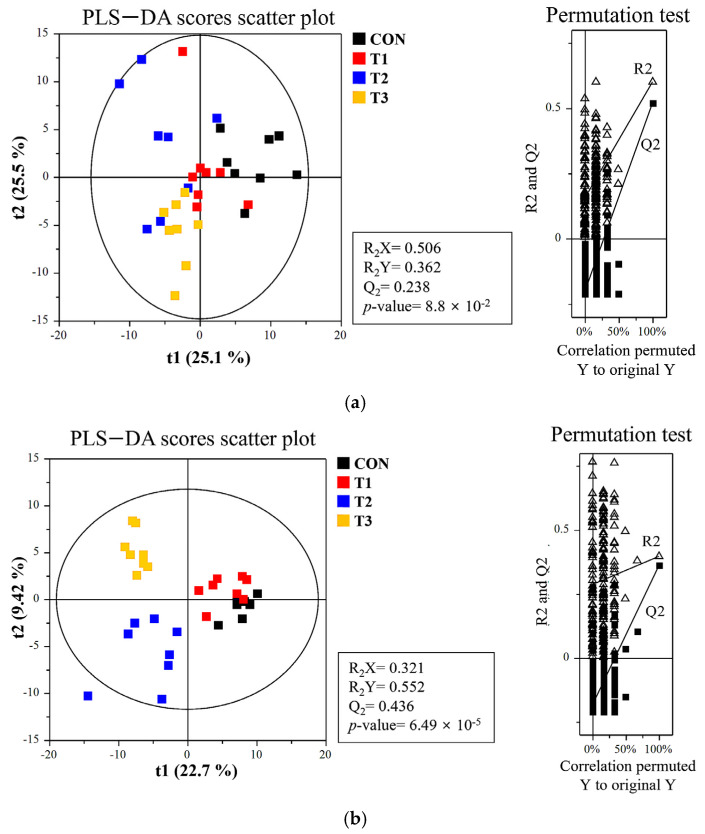

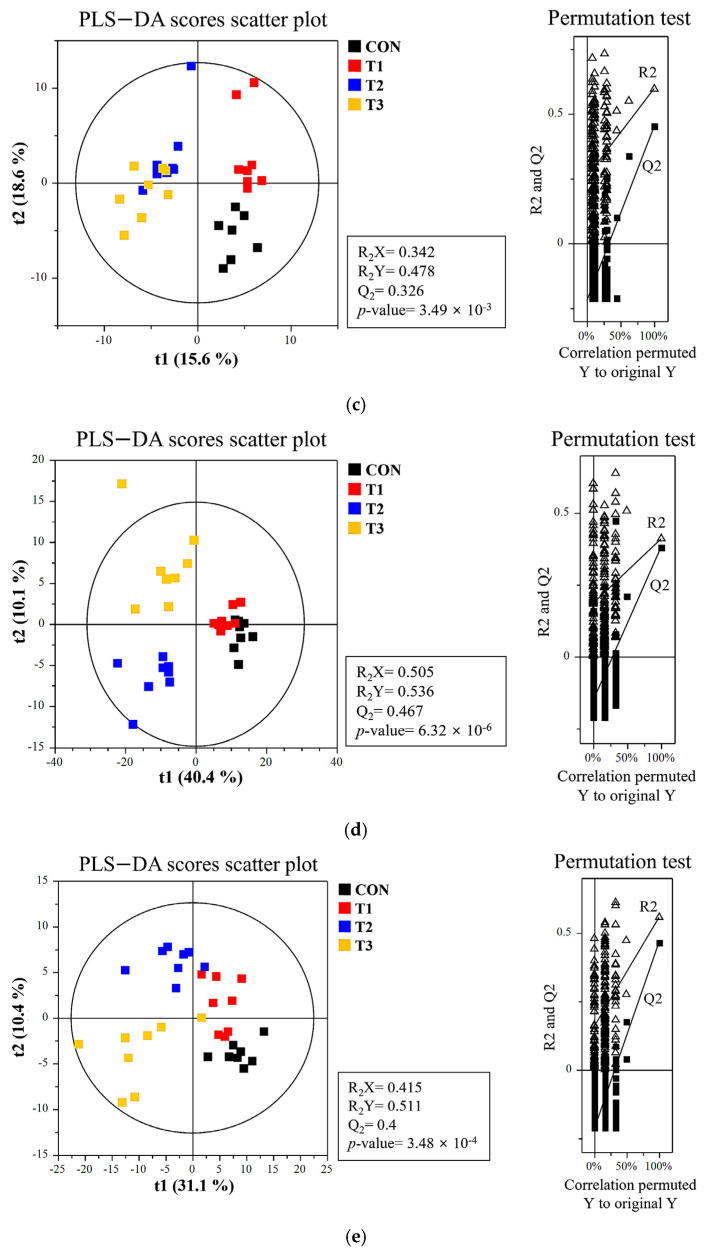

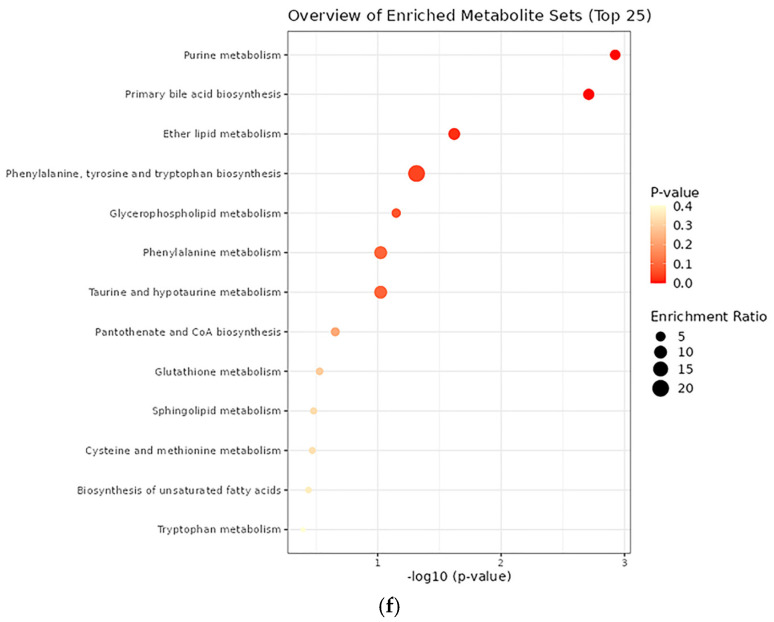
Metabolite profiling of rats subjected to different doses of deoxynivalenol (DON). Partial least discriminant analysis (PLS−DA) scatter plots and permutation plots for the (**a**) blood, (**b**) liver, (**c**) kidney, (**d**) cecum, (**e**) feces, and (**f**) metabolic pathways in different DON treatment groups. A 95% confidence interval was used to define deviations in the score plots. Clear clustering (*p* < 0.001) was observed for DON-treated groups compared with the control group. Treatment groups: CON: control group, 0.9% saline by gavage; T1: 0.5 mg/L DON with 0.9% saline by gavage; T2: 50 mg/L DON with 0.9% saline by gavage; T3: 100 mg/L DON with 0.9% saline by gavage.

We used LC-MS to profile metabolites in rat blood, liver, kidney, cecum, and feces under different DON toxicity levels. PLS-DA revealed a clear metabolic separation between DON-treated and control groups (Figure 7a–e). Metabolite enrichment was analyzed by determining significant differences in compounds between the control and DON treatment groups (variable importance projection (VIP) > 1.0, *p* < 0.05). Except for metabolites with an uncertain Kyoto Encyclopedia of Genes and Genomes (KEGG) structure, the DON-treated groups were mainly enriched in terms of metabolism of purine, followed by changes in the biosynthesis of the primary bile acid, ether lipid metabolism, and phenylalanine, tyrosine, and tryptophan biosynthesis (Figure 7f; false discovery rate (FDR) < 1, *p* < 0.05).

**Figure 8:**
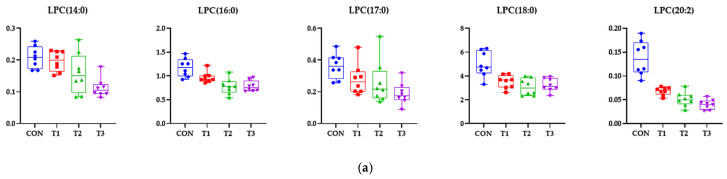

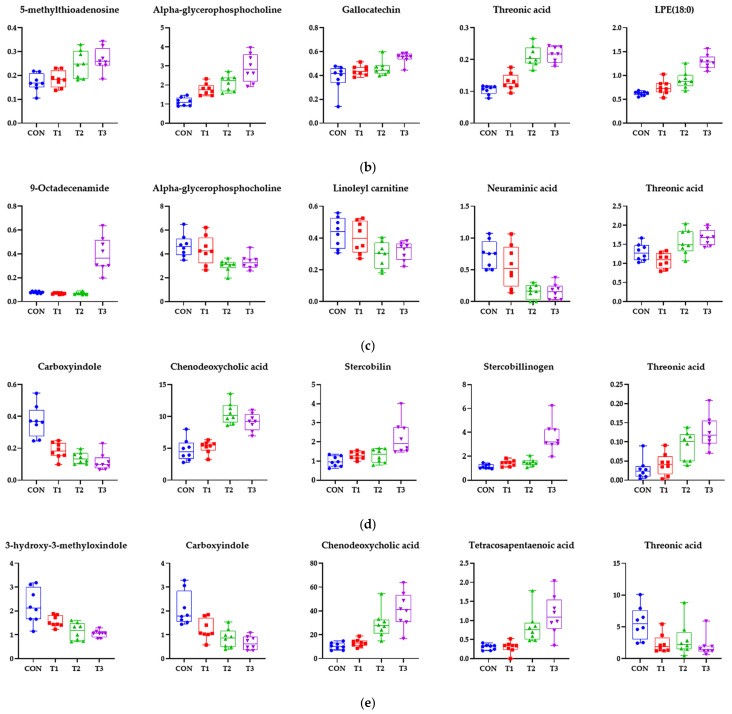
Representative bar graphs of important variables of metabolite projection in the blood and four different tissues: (**a**) blood, (**b**) liver, (**c**) kidney, (**d**) cecum, and (**e**) feces. The metabolites in the different samples were significantly different, as determined using an ANOVA model based on Tukey’s test for comparison of means. Treatment groups: CON: control group, 0.9% saline by gavage; T1: 0.5 mg/L deoxynivalenol (DON) with 0.9% saline by gavage; T2: 50 mg/L DON with 0.9% saline by gavage; T3: 100 mg/L DON with 0.9% saline by gavage.

Additional statistical analyses for DON-treated rats revealed tissue-specific biomarkers. Metabolites from the blood, liver, kidney, cecum, and feces showed significant alterations with VIP scores > 1.0 and *p* < 0.05. Notably, in the blood, different LPC species (i.e., LPC(14:0), LPC(16:0), LPC(17:0), LPC(18:0), and LPC(20:2)) were significantly decreased in the DON groups compared with the control group (Figure 8a). In the liver, 5-methylthioadenosine, alpha-glycerophosphocholine, gallocatechin, threonic acid, and LPE(18:0) were significantly increased in the treatment groups (Figure 8b). In the kidney, alpha-glycerophosphocholine, linoleyl carnitine, and neuraminic acid were significantly reduced by DON treatment. However, 9-octadecenamide and threonic acid were significantly increased by the DON diet (Figure 8c). In the cecum, chenodeoxycholic acid, stercobilin, stercobilinogen, and threonic acid were significantly increased in the DON groups compared with the control group, but carboxyindole was not (Figure 8d). In the feces, 3-hydroxy-3-methyloxindole, carboxyindole, and threonic acid were significantly decreased by DON treatment, but chenodeoxycholic acid and tetracosapentaenoic acid were significantly increased by the DON diet (Figure 8e). 

### 3.8. Simple Linear Regression Analysis

**Figure 9:**
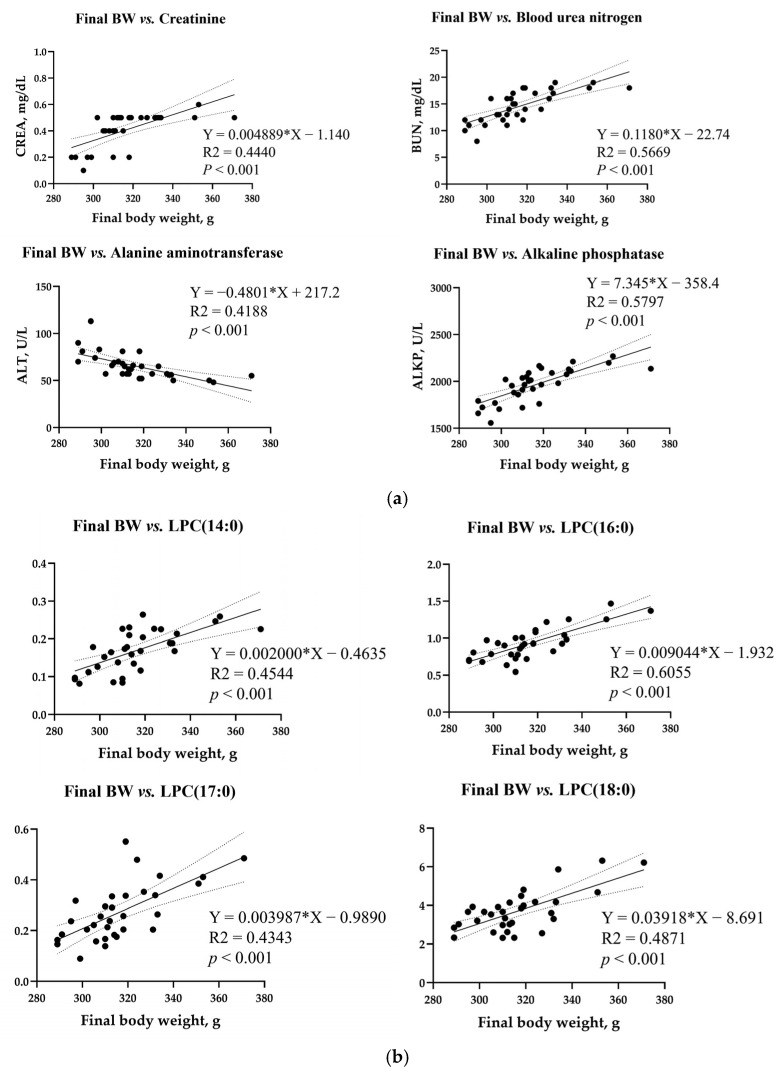

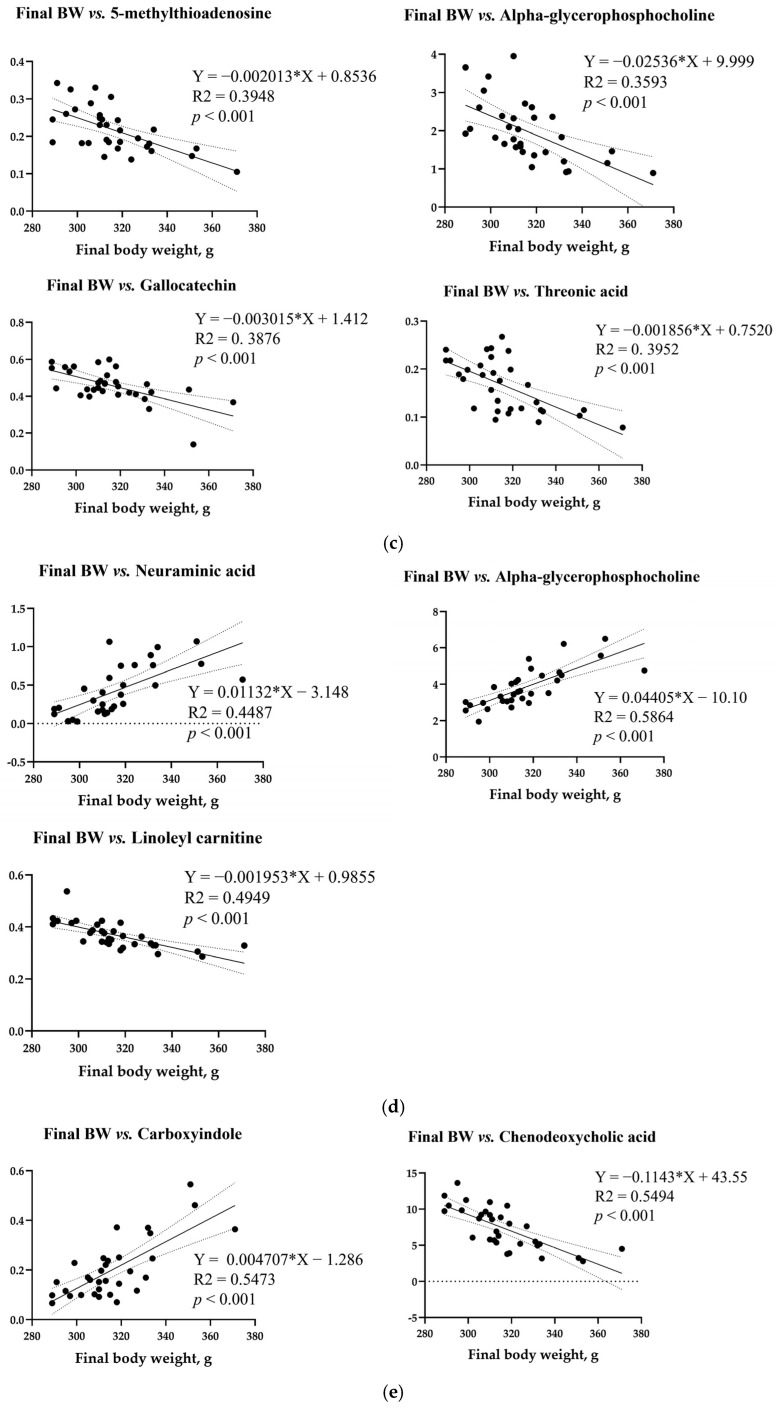

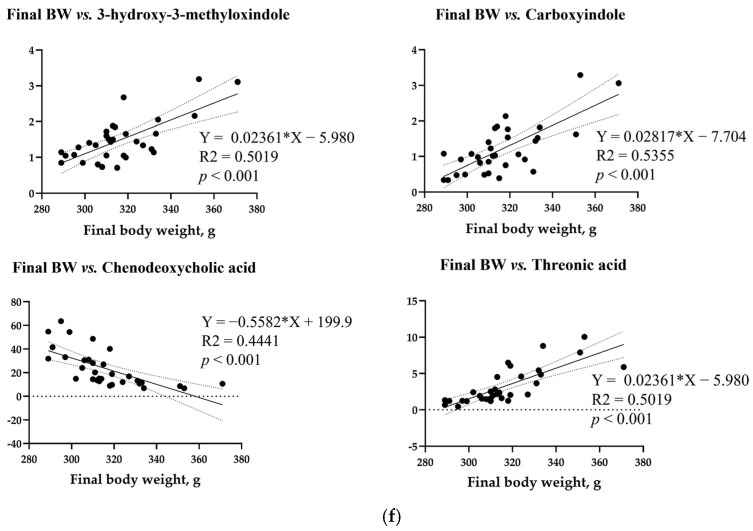
Simple linear regression analysis of the correlation of final body weight with biochemical parameters and metabolite compounds in all the treatment groups. Correlations of final body weight with (**a**) blood parameters and metabolites in the (**b**) blood, (**c**) liver, (**d**) kidney, (**e**) cecum, and (**f**) feces. Correlation coefficients and *p*-values were calculated using GraphPad Prism (ver. 9.5.1). Linear analysis conditions: variable importance projection (VIP) > 1, *p* < 0.05, R^2^ > 0.3.

Simple linear regression analysis was performed for the final rat body weight, blood biochemical parameters, metabolites, and microbiota with VIP > 1 and *p* < 0.05 (Figure 9). A simple linear regression was shown with R^2^ > 0.3. Among the biochemical parameters, CREA (R^2^ = 0.4440, *p* < 0.001), BUN (R^2^ = 0.5669, *p* < 0.001), and ALKP (R^2^ = 0.5797, *p* < 0.001) showed a positive correlation with final body weight, whereas the ALT activity in the blood (R^2^ = 0.4188, *p* < 0.001) showed a negative correlation with final body weight (Figure 9a). Among the metabolites, blood LPC(14:0) (R^2^ = 0.4542, *p* < 0.001), LPC(16:0) (R^2^ = 0.6055, *p* < 0.001), LPC(17:0) (R^2^ = 0.4343, *p* < 0.001), and LPC(18:0) (R^2^ = 0.4871, p < 0.001) showed a positive correlation (Figure 9b). In the liver, 5-methylthioadenosine (R^2^ = 0.3948, *p* < 0.001), alpha-glycerophosphocholine (R^2^ = 0.3593, *p* < 0.001), gallocatechin (R^2^ = 0.3876, *p* < 0.001), and threonic acid (R^2^ = 0.3952, *p* < 0.001) were negatively correlated with final body weight (Figure 9c). In the kidney, neuraminic acid (R^2^ = 0.4487, *p* < 0.001) and alpha-glycerophosphocholine (R^2^ = 0.5864, *p* < 0.001) levels increased with body weight. On the contrary, the level of linoleyl carnitine (R^2^ = 0.4949, *p* < 0.001) was inversely proportional to the body weight (Figure 9d). The cecum metabolite carboxyindole (R^2^ = 0.5473, *p* < 0.001) showed a positive correlation, but chenodeoxycholic acid (R^2^ = 0.4487, *p* < 0.001) exhibited a negative correlation (Figure 9e). In the feces, 3-hydroxy-3-methyloxindole (R^2^ = 0.5019, *p* < 0.001), carboxyindole (R^2^ = 0.5355, *p* < 0.001), and threonic acid (R^2^ = 0.5019, *p* < 0.001) levels increased with body weight, whereas the levels of chenodeoxycholic acid (R^2^ = 0.4441, *p* < 0.001) correlated inversely with body weight (Figure 9f). No correlation was found between the final body weight and the total microbial community, with the R^2^ value being 0.3 or higher.

### 3.9. Functional Prediction for Cecal and Fecal Microbiota

**Figure 10:**
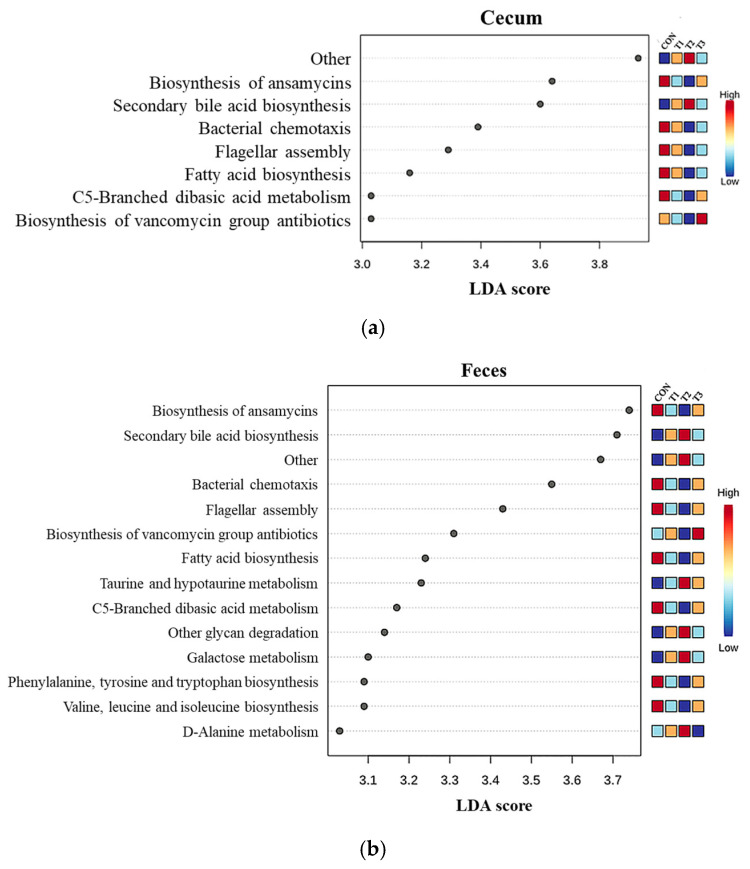
Functional predictions for cecal and fecal microbiota using PICRUSt2 analysis. (**a**) Cecum and (**b**) feces samples from the four groups were analyzed. The pathways were predicted based on the total possible number of genes (according to the KEGG database). All pathways were significant according to LEfSe. A dot plot was used to compare the four different samples, and only predicted functions with *p* < 0.05 are shown.

The potential of microbial communities to function was assessed using the PICRUSt2 method based on the KEGG database, using the ASV table of QIIME2 from the cecum and feces of DON-treated rats (Figure 10). A combination of LDA and LEfSe revealed important features in the cecal and fecal samples by comparing and identifying significantly different predictive functions in rats. Seven significant functions were predictive for the cecum (Figure 10a). Among them, secondary bile acid biosynthesis was higher in T2, and the biosynthesis pathway of vancomycin group antibiotics, which was included in the KEGG pathway, was higher in T3 than in the control group (*p* < 0.01). In addition, fatty acid biosynthesis, ansamisin biosynthesis, flagellar assembly, bacterial chemotaxis, and C5 branch dibasic acid metabolism were lower in the DON-treated group than in the control group. When comparing feces, 14 significant predictions were obtained (Figure 10b). Taurine and hypo-taurine metabolism, secondary bile acid biosynthesis, galactose metabolism, other glycan degradation, and D-alanine metabolism were higher in the T2 group, whereas the biosynthetic pathway of vancomycin group antibiotics was higher in the T3 group than in the control group (*p* < 0.01). Fatty acid biosynthesis, phenylalanine, tyrosine, tryptophan biosynthesis, flagellar assembly, ansamisin biosynthesis, valine biosynthesis, leucine biosynthesis, isoleucine biosynthesis, bacterial chemotaxis, and C5 branched-chain dibasic acid metabolism in the DON-treated group had lower levels among these pathways (*p* < 0.01). Comparing the contents of the cecum and feces, 21 KEGG pathways, including fatty acid biosynthesis, flagellar assembly, ansamycin biosynthesis, bacterial chemotaxis, and C5 branched-chain dibasic acid metabolism, were predicted to be relatively higher in the control group. Secondary bile acid biosynthesis and vancomycin group antibiotic biosynthesis were higher in T2 and T3, respectively.

## 4. Discussion

This study evaluated the toxic effects of 42-day exposure to DON at doses of 0.5, 50, and 100 ppm on growth, blood chemistry, histopathology, gut microbiota, and metabolic profiles in 7-week-old rats, and revealed that its effects differed with the dose administered [16,21]. We used a minimum treatment dose (T1) of 0.5 ppm, which is lower than the 1 ppm recommended by the Food and Drug Administration (FDA) for finished wheat products intended for human consumption [22]. In a 10-year survey, the highest DON concentration detected in 59,107 samples, including finished feed, corn, corn distillers’ dried grains with solubles, corn silage, soybean grain, soybean meal, wheat, barley, and rice, was approximately 85 ppm [5]. We accordingly set the maximum treatment level (T3) at 100 ppm and the intermediate treatment level (T2) at 50 ppm. Extreme concentrations (>100 ppm) can help us to understand the potential acute and chronic effects of DON, although they may not occur in natural environments.

Several studies have demonstrated that DON negatively affects the growth performance of rats [12,18]. In the present study, when DON was orally administered to 7-week-old rats for 42 days, the final body weight decreased by 7.1% in the T1 group, 7.7% in the T2 group, and 11.9% in the T3 group, compared with that in the control group. Additionally, compared with the control group, the ADG decreased by 17.6% in the T1 group, 19.4% in the T2 group, and 28.6% in the T3 group. Furthermore, compared with the control group, the FCR increased by 20.5% in the T1 group, 20.7% in the T2 group, and 29.7% in the T3 group. The DON-induced decrease in growth performance was dose-dependent. Growth retardation is a major side effect of DON intake, and in livestock, it can lead to significant economic losses because of its accompanying symptoms, such as diarrhea, vomiting, and reduced feed intake [23,24]. However, DON did not affect the ADFI among the treatment groups. Approximately 85% of weight loss resulting from mycotoxicosis is believed to be due to reduced feed consumption [25]. DON not only reduces feed intake, but also damages the intestinal lining—impairing nutrient absorption—and disrupts organ function, leading to reduced nutrient efficiency [26,27]. Thus, further research on the multiple effects of DON is needed to clarify the exact cause of weight loss.

In this study, oral administration of DON to rats significantly affected the serum levels of BUN and CREA, and the activities of AMYL, ALT, and ALKP. AMYL is a digestive enzyme that converts glycogen into maltose and glucose, supplying essential energy to the body [28]. However, ingesting DON damages the intestinal mucosa, increases permeability, and thereby reduces nutrient absorption while impairing digestive organ function [29]. This damage may ultimately inhibit the secretion of digestive enzymes such as AMYL. BUN and CREA are by-products of protein and creatinine metabolism, respectively [30]. These two parameters are important indicators of the severity of kidney damage, as they reflect the degree of glomerular filtration [30]. Therefore, our results suggest that the kidneys of the rats were significantly damaged by high levels of DON. Both ALT and ALKP are reliable parameters for assessing liver damage [31,32]. The liver is particularly vulnerable to DON toxicity, because it is the primary organ responsible for detoxifying and metabolizing DON after its absorption [33]. Consistent with our findings, Pinton et al. [26] reported increased blood ALT levels in DON-treated rats, whereas Gerez et al. [12] found decreased blood ALKP levels in DON-treated rats. Thus, the changes in blood ALT and ALKP levels observed in the DON-treated group may indicate liver damage due to DON-induced systemic toxicity resulting from abnormal excretion of hepatic metabolites [34].

We found that higher doses led to more extensive apoptosis and fibrosis in organs such as the liver, kidney, muscle, jejunum, and adipose tissue. These findings are similar to those of our previous studies and other studies in rats [13,35]. The histological changes observed in the kidney, liver, and small intestine may explain the abnormal secretion of serum AMYL, BUN, CREA, ALT, and ALKP described above. DON is largely absorbed by the body upon ingestion and is rapidly distributed to all tissues, mainly the blood, gastrointestinal tract, lymphatic system, and immune system [10]. As a result, it can adversely affect various organs. Although the precise mechanism is not fully clear, DON is known to cause nephrotoxicity characterized by renal dysfunction, oxidative stress, and apoptosis [30]. Due to its central role in detoxifying and metabolizing mycotoxins, the liver is particularly vulnerable to DON toxicity. DON alters the structural conformation of the liver, induces oxidative stress, and promotes apoptosis [33,36]. The small intestine is a site of intensive mycotoxin absorption and metabolism, and the jejunum is apparently susceptible to DON toxicity [37]. Oxidative stress-mediated effects could be the mechanism underlying the DON-induced histological changes observed in various organs.

Several studies have indicated that DON induces oxidative stress via the generation of ROS [38,39]. Moreover, it alters the antioxidant defense systems in various target tissues by impairing the functions of key antioxidant enzymes, such as superoxide dismutase, gamma-glutamyltransferase, glutathione S-transferase, and catalase [40]. Liang et al. [30] showed that DON significantly increased the apoptosis rate, while decreasing the superoxide dismutase activity and hydroxyl radical (OH-) inhibition ability and increasing the malondialdehyde content, in mouse kidneys. Ji et al. [38] found a close correlation between the levels of malondialdehyde and 8-hydroxy-2′-deoxyguanosine, which are indicators of oxidative damage, and the number of apoptotic cells in porcine liver, demonstrating that apoptosis of hepatocytes is induced by DON-mediated oxidative damage. DON-induced oxidative stress was found to upregulate apoptotic and inflammatory markers, including interleukin-1 beta (IL-1β), cyclooxgenase-2 (COX-2), interleukin-6 (IL-6), tumor necrosis factor-alpha (TNF-α), and caspases 3, 8, and 9, in porcine intestinal epithelial cells (IPEC-J2 cells) [37].

Fibrosis is defined as the excessive accumulation of fibrotic connective tissue in the extracellular matrix of damaged tissue [41]. Oxidative stress boosts fibrotic factors such as TGF-β1, leading to fibrosis because of the accumulation of extracellular matter [41,42]. Moreover, oxidative stress can trigger a release of inflammatory cytokines, such as TNF-α, monocyte chemoattractant protein (MCP-1), IL-6, and IL-8, which can cause tissue fibrosis [41,42,43]. Our results demonstrate that dose-dependent increases in DON have profound effects on multiple organs in the body. However, we did not analyze DON-induced oxidative stress to verify this hypothesis. Therefore, future studies should investigate histological changes, including fibrosis, apoptosis, and DON-induced oxidative stress.

The gut microbiome is an important factor influencing inflammation, immunity, and disease in the host [44]. The intestinal microbiota is essential for animal health, offering nutritional benefits and protecting against pathogens, while maintaining effective mucosal immunity. The gut is the first physiological barrier to DON exposure. Toxicants can disrupt gut homeostasis by inducing gut damage, inflammation, and dysbiosis of the gut microbiota [45]. Rats are commonly used as an in vivo model for studying the gut microbiome, elucidating the relationship between gut microbiota dysbiosis and disease, and for analyzing the toxic effects of mycotoxins on the microbiome [46,47]. In this study, beta diversity analysis using the Bray–Curtis index revealed distinct clustering in both the cecal and fecal microbiomes of the treatment groups, suggesting that DON exposure significantly altered the microbial composition. In addition, alpha diversity metrics (ACE, Shannon, Simpson) showed significant differences at both sites, although the patterns varied, likely due to regional differences in gut conditions (e.g., nutrient content, pH, water, oxygen, and temperature) [48]. In addition, microbial communities are affected by environmental conditions and biological interactions [46,47]. In this regard, our study investigated the microbial diversity in both the cecal tract and fecal samples. At the phylum level, Firmicutes and Bacteroidota were the predominant phyla in both the cecum and feces, which is consistent with previous studies on rats [49,50]. High doses of DON (≥50 ppm) increased the abundance of Proteobacteria in both the cecum and colon. An increase in Proteobacteria can be used as an indicator of dysbiosis development, and is positively correlated with colitis and metabolic disorders [51]. The abundance of Firmicutes in the cecal microbiota was decreased upon DON exposure. Firmicutes facilitate energy metabolism in the host by converting complex carbohydrates into short-chain fatty acids, and an increase in the abundance of this phylum has been associated with weight gain in mice [48,52]. In contrast, DON exposure increased the abundance of Bacteroidota in the cecal microbiota. Bacteroidota is a major phylum in the mouse gut microbiota, but its increased abundance can lead to weight loss [53]. High doses of DON (≥50 ppm) also increased the abundance of Desulfobacterota in the rat cecal microbiota and increased the abundance of Actionobacteria in the fecal microbiota in a pregnant rat model. Elevated levels of Desulfobacterota can cause stress and depression, and suppressing them improves movement disorders and neurological deficits [54]. Increased abundance of Actinobacteria was found to be associated with energy loss via the feces. Additionally, the control and low-dose DON (0.5 mg/L) groups showed increased abundance of Campylobacterota. Campylobacterota may play a positive role in food breakdown and digestion [55]. However, because certain species (*Campylobacter jejuni* and *Helicobacter pylori*) belonging to the phylum Campylobacterota are generally known as harmful pathogens, further investigation is deemed necessary [56,57].

Few studies have analyzed the gut microbiota at the species level to evaluate the toxic effects of DON in rats. In the present study, *Lactobacillus acidophilus*, *Romboutsia ilealis*, *Lactobacillus reuteri*, *Lactobacillus murinus*, and *Lactobacillus intestinalis* were the top five predominant species in the cecum and feces, with *Lactobacillus acidophilus* being the most predominant. The LEfSe analysis indicated that *Lactobacillus acidophilus* was the least abundant in the cecum and feces of rats in the control group. *Lactobacillus acidophilus* influences the composition of the gut microbiota, enhances immune function, and improves nutrient absorption [58]. However, feeding certain *Lactobacillus acidophilus* strains to mice increased the abundance of harmful bacteria and exacerbated colitis in another study [59]. Nevertheless, because the efficacy of this microorganism has been demonstrated in several studies and it is considered a probiotic, further research is needed to elucidate the precise mechanism underlying these findings. A decrease in the abundance of *Bacteroides vulgatus* was observed in the feces of control group rats. However, some studies have shown that the abundance of *Bacteroides vulgatus* is higher in patients with ulcerative colitis, and certain strains induce the expression of inflammatory cytokines [60]. In this study, *Romboutsia ilealis* had the highest relative abundance in the feces of rats in the T3 group; this microorganism is pathogenic and is associated with metabolic diseases, such as diabetes and obesity [61].

The PICURSt2 results showed 7 and 14 important predictive functions in the cecum and feces, respectively. These included bile acid biosynthesis, fatty acid biosynthesis, flagellar assembly, ansamycin biosynthesis, bacterial chemotaxis, and C5 branched-chain dibasic acid metabolism. Therefore, cells may be involved in establishing an integrated stress response system by remodeling cell membranes, regulating energy balance, and activating protective metabolism in response to DON toxicity. DON increases calcium release by damaging cell membranes or disrupting bone cell function. This can lead to lipid peroxidation and osteoporosis. Therefore, DON can weaken the antioxidant defense and increase oxidative stress associated with lipid metabolism [62]. Ansamycin is a polyketide antibiotic that can respond to the effects of toxins or play a protective role by regulating stress signals through conversion to secondary metabolic pathways, such as the biosynthesis of ansamycin by DON. Ansamitocin targets Hsp90, inhibiting proteins vital to the growth and survival of cancer cells. This prevents cell division, inducing apoptosis. Some ansamycin derivatives also bind to tubulin, affecting cell division, which makes them a promising anticancer drug [63]. DON is expected to cause biological changes by affecting this metabolic pathway. The scientific understanding of how cells coordinate and respond will be improved by obtaining mechanistic insights into the metabolic pathways of DON toxins.

In the present study, we conducted a metabolomic analysis to investigate the biological pathways affected by DON toxicity across a range of low to high doses. Metabolites are the final products or intermediates of cellular activities, and reflect the overall response of organs or biological systems under different pathophysiological conditions [64]. Understanding the metabolic pathways affected by DON could provide valuable information for risk assessment, prevention, and management [2]. Our results showed distinct metabolic profiles in the blood, liver, kidney, cecum, and feces, revealing clear differences between the control and DON groups. These findings are consistent with our previous study, wherein we examined the effects of low-dose DON (0.02 and 0.2 ppm) in rats [13]. Low-dose DON affected lipid metabolism, glycerophospholipid metabolism, and phenylalanine metabolism; among the metabolites, phenylalanine levels in the blood and feces were reduced. Phenylalanine is an essential amino acid that is metabolized by intestinal bacteria [65]. Threonic acid is a metabolite of ascorbic acid (vitamin C), and plays an important role in gut health and oxidative stress management [66]. DON can affect this metabolic process, potentially altering the intestinal environment and *Campylobacter* survival [67]. Low doses of DON can disrupt the balance of the gut microbiome and promote the growth of *Campylobacter*, particularly *C. jejuni*. This is expected to affect gut health by altering the metabolism of essential amino acids (e.g., phenylalanine and threonic acid). Interactions between microbes, metabolism, and the environment, such as DON, are complex, and may influence the survival and proliferation of cells. To identify metabolic biomarkers related to growth under DON toxicity, we analyzed the correlation between metabolites driving the separation among dietary treatment groups and final body weight. Using this method, it is possible to predict body weight based on changes in metabolites. Uric acid, an oxidative by-product of purine metabolism, regulates metabolism in the liver, adipose tissue, and muscle. Hyperuricemia may be associated with diseases such as metabolic syndrome and fatty liver [68]. Bile acids are synthesized from cholesterol in the liver and combined with glycine or taurine to increase their water solubility. They are then absorbed in the small intestine and recirculated to the liver; only a small amount is excreted via feces. Gut microbes convert primary bile acids into secondary bile acids, affecting toxicity and metabolic pathways. Bile acids play an important role as metabolic signaling molecules, and their metabolic pathways impact health and disease [69]. Bile acids regulate metabolism and influence inflammation. Changes in bile acid metabolism are associated with diseases, such as cardiovascular disease, non-alcoholic fatty liver disease, diabetes, and cancer [70]. We expect that bile acid research will help to elucidate and mitigate DON toxicity. Anti-tuberculosis drugs, such as rifampicin, isoniazid, and pyrazinamide, have been shown to cause hepatotoxicity in C57BL/6 mice. These hepatotoxicities have been associated with bile acid, lipid, and purine metabolism [71]. Bile acids are important for metabolism and inflammation. Abnormal bile acid metabolism can lead to heart, metabolic, and inflammatory conditions, as well as cancer. Changes in the intestinal microbiota upon DON exposure may increase the biosynthesis of secondary bile acids [72]. In addition, increased deoxycholic acid can cause damage to the intestinal barrier and reduce the expression of bile acid transporters [73]. Increases in threonic acid were associated with weight gain in rats, whereas increases in chenodeoxycholic acid correlated with weight loss. Threonic acid is the major breakdown product of ascorbate, the reduced form of vitamin C [74]. Ascorbate functions as an antioxidant by stabilizing ROS [75], which indicates that DON-induced oxidative stress may be attenuated by ascorbate metabolism. Conversely, chenodeoxycholic acid is a primary bile acid synthesized in the liver, and its excessive accumulation can cause mitochondrial damage and oxidative stress [76], indicating adverse effects from DON-induced hepatotoxicity. Therefore, our results suggest that the ascorbate metabolic pathway and bile acid synthesis may serve as growth-related biomarkers of DON toxicity.

## 5. Conclusions

This study demonstrates that oral DON gavage induced adverse effects in rats, including impaired growth performance and altered blood biochemistry, with high doses (≥50 ppm) exacerbating these toxic effects. Additionally, DON toxicity caused significant dose-dependent histological changes, such as fibrosis and apoptosis, in various organs. Notably, microbial communities in both the cecum and feces were modified by DON toxicity, with more pronounced changes observed at higher doses. These findings indicate that DON can cause health issues in rats even at levels below the maximum allowable limit, with the toxicity worsening at increased doses. Although further investigations are required, this study can be used as a basis for performing toxicity studies. The insights obtained can potentially be applied to the determination of DON contamination for the assessment of food safety and toxicology aspects.

## Data Availability

The dataset is available on request from the authors.

## Abbreviations

The following abbreviations are used in this manuscript:

ACE	Abundance-based coverage estimator
ADFI	Average daily feed intake
ADG	Average daily gain
ALKP	Alkaline phosphatase
ALT	Alanine aminotransferase
AMYL	Amylase
ANOVA	Analysis of variance
ASV	Amplicon sequence variants
BUN	Blood urea nitrogen
CON	Control
CREA	Creatinine
DON	Deoxynivalenol
EFSA	European Food Safety Authority
FCR	Feed conversion ratio
FDA	Food and Drug Administration
FDR	False discovery rate
KEGG	Kyoto Encyclopedia of Genes and Genomes
LDA	Linear discriminant analysis
LEfSe	LDA effect size
NBF	Neutral buffered formalin
PERMANOVA	Permutational multivariate analysis of variance
PLS-DA	Partial least squares discriminant analysis
SEM	Standard error of the mean
SMRT	Single Molecule Real-Time
T1	0.5 ppm DON
T2	50 ppm DON
T3	100 ppm DON
TUNEL	Terminal deoxynucleotidyl transferase dUTP nick-end labeling
VIP	Variable importance projection
